# Community Behavioral and Perceived Responses in the COVID-19 Outbreak in Afghanistan: A Cross-Sectional Study

**DOI:** 10.1017/dmp.2021.135

**Published:** 2021-05-05

**Authors:** Sayed Hamid Mousavi, Mohammad Hossein Delshad, Cecilia Acuti Martellucci, Divya Bhandari, Akihiko Ozaki, Fatemeh Pourhaji, Fahimeh Pourhaji, Sayed Mohammad Reza Hosseini, Rohullah Roien, Abass Ali Ramozi, Marzia Wafaee, Shohra Qaderi, Mohammad Delsoz, Shailendra Sigdel, Yasuhiro Kotera, Toyoaki Sawano, Kuldeep Dhama, Alfonso J. Rodríguez-Morales, Jiwei Wang, Tetsuya Tanimoto, Ali Ahmad Yousefi, Ranjit Sah

**Affiliations:** 1 Medical Research Center, Kateb University, Kabul, Afghanistan; 2 Afghanistan National Charity Organization for Special Diseases (ANCOSD), Kabul, Afghanistan; 3 Public Health Department, School of Health, Torbat Heydariyeh University of Medical Sciences, Torbat Heydariyeh, Iran; 4 Health Sciences Research Center, Torbat Heydariyeh University of Medical Sciences, Torbat Heydariyeh, Iran; 5 Department of Medical Sciences, University of Ferrara, Ferrara, Italy; 6 Medical Governance Research Institute, Tokyo, Japan; 7 Department of Breast Surgery, Jyoban Hospital of Tokiwa Foundation, Fukushima, Japan; 8 Department of Health Education and Health Promotion, School of Health, Mashhad University of Medical Sciences, Mashhad, Iran; 9 Medical Research Center, Razi Institute of Higher Education, Kabul, Afghanistan; 10 Faculty of Chemistry, Kabul University, Kabul, Afghanistan; 11 School of Medicine, Shahid Beheshti University of Medical Science, Tehran, Iran; 12 NOOR Eye-care Training Center of International Assistance Missions, Kabul, Afghanistan; 13 Department of Cardiothoracic and Vascular Anesthesiology, Maharajgunj Medical Campus, Tribhuvan University Institute of Medicine, Maharajgunj, Kathmandu, Nepal; 14 Human Sciences Research Centre, University of Derby, Derby, United Kingdom; 15 Department of Radiation Health Management, Fukushima Medical University School of Medicine, Fukushima, Japan; 16 Division of Pathology, ICAR-Indian Veterinary Research Institute, Bareilly, India; 17 Grupo de Investigación Biomedicina, Faculty of Medicine, Fundación Universitaria Autónoma de las Américas, Pereira, Colombia; 18 School of Public Health, Fudan University, Shanghai, China; 19 Tribhuvan University Teaching Hospital, Institute of Medicine, Kathmandu, Nepal

**Keywords:** perceived threat, COVID-19, perceived susceptibility, perceived severity, behavioral response, Afghanistan

## Abstract

**Objective:**

Community responses are important for the management of early-phase outbreaks of coronavirus disease 2019 (COVID-19). Perceived susceptibility and severity are considered key elements that motivate people to adopt nonpharmaceutical interventions. This study aimed to (i) explore perceived susceptibility and severity of the COVID-19 pandemic, (ii) examine the practice of nonpharmaceutical interventions, and (iii) assess the potential association of perceived COVID-19 susceptibility and severity with the practice of nonpharmaceutical interventions among people living in Afghanistan.

**Methods:**

A cross-sectional design was used, using online surveys disseminated from April to May 2020. Convenience sampling was used to recruit the participants of this study. The previously developed scales were used to assess the participants’ demographic information, perceived risk of severe acute respiratory syndrome coronavirus 2 (SARS-CoV-2) infection, and perceived severity of COVID-19. Multivariate analyses were conducted to assess the potential association of perceived COVID-19 susceptibility and severity with the practice of nonpharmaceutical interventions.

**Results:**

The Internet was the main source for obtaining COVID-19 information among participants in this study. While 45.8% of the participants believed it was “very unlikely” for them to get infected with COVID-19, 76.7% perceived COVID-19 as a severe disease. Similarly, 37.5% believed the chance of being cured if infected with COVID-19 is “unlikely/very unlikely.” The majority of participants (95.6%) perceived their health to be in “good” and “very good” status. Overall, 74.2% mentioned that they stopped visiting public places, 49.7% started using gloves, and 70.4% started wearing a mask. Participants who believed they have a low probability of survival if infected with COVID-19 were more likely to wear masks and practice hand washing.

**Conclusions:**

It appears that communities’ psychological and behavioral responses were affected by the early phase of the COVID-19 pandemic in Afghanistan, especially among young Internet users. The findings gained from a timely behavioral assessment of the community might be useful to develop interventions and risk communication strategies in epidemics within and beyond COVID-19.

The coronavirus disease 2019 (COVID-19) pandemic has taken a severe toll on the health and well-being of people globally.^1^ Studies have shown that human behaviors are more likely to influence the risk of infection with severe acute respiratory syndrome coronavirus 2 (SARS-CoV-2).^2^ While drugs and vaccines have been developed and administered to reduce the severity of COVID-19, their effectiveness to fully contain COVID-19 outbreaks is still unclear. Therefore, nonpharmacological interventions (NPIs), such as the use of masks, regular hand washing, and maintaining social distancing are mandatory, and these preventive measures have been encouraged by both authorities and health-care providers to limit the spread of the disease.^3,4^ According to the Health Belief Model (HBM) and the Protection Motivation Theory (PMT),^5^ perceived susceptibility and perceived severity are key factors in alleviating the global threat of the ongoing COVID-19 epidemic,^6^ because populations who perceive themselves at a higher risk of having health problems are more likely to engage in preventive behaviors. However, adequate and appropriate information is needed to understand specific situations and follow the precautions effectively. In this age of science and technology, risk perception toward COVID-19 can be heavily influenced by social media narratives and multiple information technology sources.^7^ For example, the overuse of mass media and social network services in communicating the fear of COVID-19 might contribute to overreaction, unwarranted public fear, and an overly pessimistic perception of the current risk.^8^ Therefore, a timely assessment of the psychological and behavioral responses of the community is critical to developing subsequent interventions and risk communication strategies as the epidemic progresses.^9^

For developing countries like Afghanistan, with a fragile health-care system, the impact of the novel COVID-19 goes far beyond the capacity of the country. The first case of SARS-CoV-2 infection in Afghanistan was a 35-y-old man identified on February 24, 2020, in Herat, the third-largest city located in the western part of the country.^10^ Despite timely initiation and implementation of containment/precautionary measures and active surveillance (when the country had a relatively low caseload of 50), the number of cases grew rapidly. As of April 1, 2021, there were 56,517, cases and 2489 deaths confirmed in Afghanistan.^11^ Although containment measures were initiated asking people to stay at home, this was not feasible for many people with financial constraints. Furthermore, unlike other high-income countries, the Government of Afghanistan was not able to provide financial assistance, leaving people with no choice but to work.^12^ In the long run, this arguably applies to all countries irrespective of their income status. Therefore, as mentioned above, it is crucial to be informed about the severity of the disease and strictly follow preventive measures to curb the spread of the virus. This study aimed to (i) explore perceived susceptibility and perceived severity of the COVID-19 pandemic (ii) examine the practice of nonpharmaceutical interventions, and (iii) assess the potential association of perceived COVID-19 susceptibility and severity with the practice of nonpharmaceutical interventions among people living in Afghanistan.

## Methods

### Setting and Participants

A cross-sectional online survey was performed from April 20, 2020, to May 25, 2020. Survey link was disseminated among the general population using different social media networks. Convenience sampling was used to recruit the participants. Anyone with the link was able to fill the survey. However, to prevent multiple responses from the same person, the survey link was designed so that a response can be provided only once from the same electronic device. Support was provided by multiple universities from 23 different provinces for data collection; however, the majority of the responses were from Kabul (the capital city of Afghanistan).

Eligible participants in this study were the general population (i) aged 17-y-old or above, (ii) who could understand Dari Persian language, and (iii) have lived in Afghanistan for at least 1 mo before the survey.

### Variables

#### Perceived Threat Assessment

The perceived threat of COVID-19 was measured through a survey section with 2 dimensions: (1) perceived susceptibility, and (2) perceived severity.

Perceived susceptibility was assessed by the questions “How likely are you to become infected with coronavirus without preventive behaviors?” and “How likely is your family to become infected with coronavirus without preventive behaviors?”. Responses were measured on a 5-point Likert scale (1 = very unlikely to 5 = very likely).

Similarly, perceived severity was measured by the questions “How serious do you believe COVID-19 is?”, “What are your chances of having COVID-19 cured?”, and “What are your chances of survival if infected with COVID-19?”. Those questions were answered on a 5-point Likert scale (1 = very low to 5 = very high). Overall scores were calculated as the sum of all answers for both perceived susceptibility (scores from 2 to 10) and perceived severity (scores from 3 to 15).

### COVID-19 Information Source

Participants were asked to report the type of source they used to obtain COVID-19 information. Options included: television/radio, doctors/nurses, families or friends, newspaper and magazines, internet, unofficial websites (including Telegram, WhatsApp, and similar apps), Facebook, and books.

They were further asked about the type of information that they were interested in receiving. Multiple choices were allowed.

### Preventive Measures Assessment

Participants were asked about their compliance with precautionary measures including hygienic practices, social distancing, and travel avoidance.

### Sociodemographic Variables

The following socio-demographic information was asked: sex, age, education, and occupational status, self-perceived health status, and travel history in the past month.

### Statistical Analysis

Of 500 responses received from the online survey, 50 were removed from analysis due to incomplete data. Collected data were checked for errors and analyzed using STATA 15.1 (StataCorp. 2017. College Station, TX). Simple descriptive statistics were presented using frequency and percentage. Multivariate logistic regression was used to assess the potential association of perceived COVID-19 susceptibility and severity with 5 behavioral outcomes (avoiding public transportation, restaurants, public places, wearing masks, and washing hands). Wearing gloves was eliminated in favor of hand washing, in view of the superiority of the second in terms of disease prevention efficacy.^13^ Odds ratios (ORs), with 95% confidence intervals (95% CI), were calculated, both unadjusted (OR) and adjusted a priori for age, sex, and educational level (AOR). Statistical significance was set as a 2-sided *P* < 0.05 for all tests, and ORs were only reported if they reached this significance level.

## Results

A total of 450 responses were included in the study for analysis. Table 1 demonstrates the socio-demographic characteristics of the participants. The majority of participants (65.8%) were in the age range from 17 to 26 y old, and more than half (71.6%) were male. Additionally, 61.8% of participants were single, and 79.1% had completed their bachelor’s degree and above level of education.

**Table 1.** Socio-demographic characteristics of participants (*N* = 450)

Variables	*n*	%
Age (y)		
17-26	296	65.8
27-36	103	22.9
37-46	37	8.2
47-56	10	2.2
57 and above	4	0.9
Sex		
Male	322	71.6
Female	128	28.4
Marital status		
Single	278	61.8
Married	168	37.3
Divorcee and widow	4	0.9
Education		
Elementary and lower	13	2.8
Guidance	22	4.9
Seminary	7	1.6
Diploma	52	11.6
Graduate	279	62.0
Postgraduate	77	17.1

Table 2 shows the perceived health status and practice of healthy behavior of the participants. More than half of the participants (58.9%) perceived their health status to be “very good.” Only 2.0% perceived their health status as being “bad” and “very bad,” and 12.4% reported having respiratory symptoms in the past 14 d.

**Table 2.** Perceived health status and practice of healthy behavior (*N* = 450)

Variables	n	%
Self-perceived health status		
Very good	265	58.9
Good	165	36.7
Middle	11	2.4
Bad/ Very bad	9	2.0
Medical visit in the past 14 days		
Yes	47	10.4
No	403	89.6
Having had respiratory symptoms in the past 14 days		
Yes	56	12.4
No	394	87.6
Past month’s travel history		
Yes	73	16.2
No	377	80.8
Behavioral outcomes		
Avoid public transportation	304	67.6
Avoid eating in restaurants	398	88.4
Avoid visiting public places	334	74.2
Wearing masks	317	70.4
Using gloves	219	49.7
Hand washing	64	14.2

Regarding the practice of healthy behavior, 88.4% of participants reported that they avoided eating in restaurants. Furthermore, 74.2% mentioned that they avoided visiting public places, 49.7% had started using gloves. The majority of participants (70.4%) also started wearing a mask.

Table 3 demonstrates risk perception (perceived susceptibility to and severity) toward COVID-19. The mean scores of perceived susceptibility and severity were 4.1 (SD 2.2) and 11.0 (SD 1.8), respectively. A total of 45.8% of the participants believed it was “very unlikely” for them to get infected with COVID-19. However, 76.6% of participants perceived COVID-19 as a severe disease. While 9.3% believed the chance of being cured if infected with COVID-19 was “very unlikely,” 18.0% believed the chance of survival if infected with COVID-19 was “likely” or “very likely.”

**Table 3.** Risk perception toward COVID-19 (*N* = 450)

Risk perception items	Mean (SD)	Number (%)				
		Level 1	Level 2	Level 3	Level 4	Level 5
Perceived susceptibility (range 0 to 10)	4.1 (2.2)					
How likely are you to become infected with the coronavirus?^a^		27 (6.0)	29 (6.4)	110 (24.4)	78 (17.3)	206 (45.8)
How likely is your family to become infected with the coronavirus?^a^		16 (3.6)	36 (8.0)	90 (20.0)	98 (21.8)	210 (46.7)
Perceived severity (range 0 to 15)	11.0 (1.8)					
What is the degree of severity of the COVID-19 disease?^b^		9 (2.0)	13 (2.9)	16 (3.6)	67 (14.9)	345 (76.6)
Chance of having COVID-19 cured?^a^		52 (11.6)	62 (13.8)	167 (37.1)	127 (28.2)	42 (9.3)
Chance of survival if infected with COVID-19?^a^		22 (4.9)	59 (13.1)	166 (36.9)	163 (36.2)	40 (8.9)

Abbreviation: SD, standard deviation.

a Level 1 = Very likely; Level 2 = Likely; Level 3 = Neutral; Level 4 = Unlikely; Level 5 = Very unlikely.

b Level 1 = Very low; Level 2 = Low; Level 3 = Neutral; Level 4 = High; Level 5 = Very high.

Table 4 shows the types of COVID-19 information that participants want to receive. Almost all (96.2%) participants were interested to know about the interventions conducted by the Afghanistan Ministry of Public Health to mitigate COVID-19. Similarly, 88.7% of participants wished to know about the actions taken by international organizations to combat COVID-19. More than half (65.6%) were interested in the signs and symptoms of COVID-19 and 63.8% in the risk and consequences of COVID-19.

**Table 4.** COVID-19 information that participants want to receive (*N* = 450)

Information you wished to receive about COVID-19	Number (%)
Interventions of Afghanistan Ministry of Public Health	433 (96.2)
Actions of international organizations to combat COVID-19	399 (88.7)
Number of patients infected with coronavirus	373 (82.9)
What to do if infected with COVID-19	343 (76.2)
Preventive proceedings	302 (67.1)
Symptoms/how to know if someone is suffering from COVID-19	295 (65.6)
The risks and consequences of COVID-19	287 (63.8)
Distribution of COVID-19 disease cases	264 (58.7)
Impact of the disease in high-risk groups	257 (57.1)
Disease advancement	241 (53.6)

**Figure 1.**
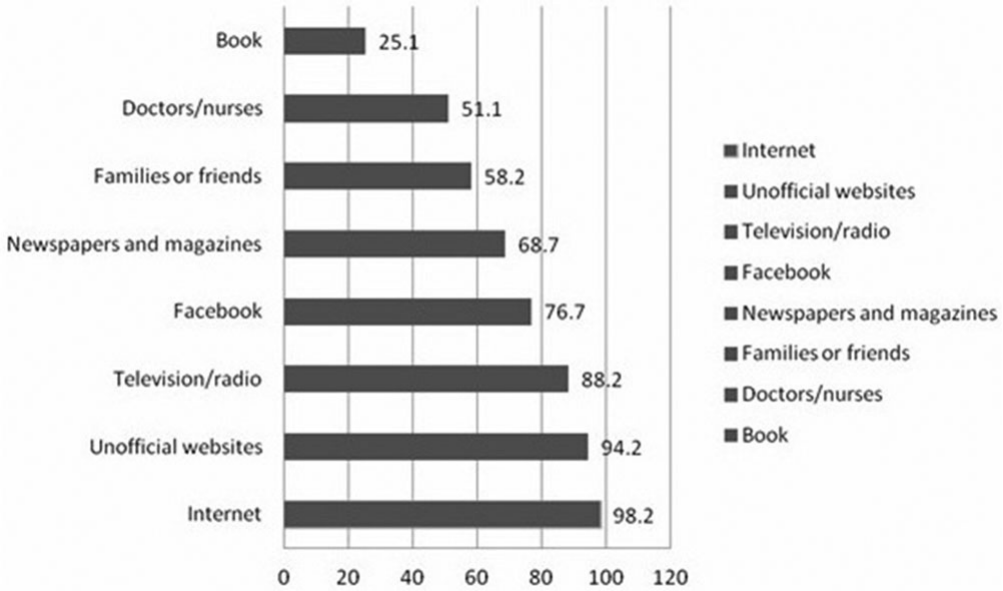
Sources used to seek information about COVID-19.

Figure 1 illustrates the sources used to seek information about COVID-19. The Internet was found to be a predominant source, with 98.2% of participants using it to seek information related to COVID-19, followed by unofficial websites (94.2%) and TV/radio (88.9%).

Table 5 shows the association of perceived COVID-19 susceptibility and severity with 5 behavioral outcomes. Believing to have a low likelihood to be cured from COVID-19 was not statistically significantly associated with any of the outcomes. Unexpectedly, the AOR of avoiding public places was 0.53 (95%CI 0.29-0.97) for responders who self-identified as being highly likely of being infected with SARS-CoV-2. Because of the cross-sectional design, it is possible that this finding was driven by reverse causality: people who reported having a higher probability of infection were those who could not avoid public places. Responders with perceived high susceptibility to infection of their family had AOR 3.60 (1.86-6.99) of practicing hand washing. Also, responders who believed to have a low probability of surviving if they fell ill with COVID-19 had AOR 1.87 (1.02-3.41) of wearing a mask, and AOR 2.01 (1.08-3.73) of practicing hand washing. Finally, avoiding public transportation and restaurants was not statistically significantly associated with any of the investigated beliefs on susceptibility and severity (except the unadjusted analysis on the possible association between a high perceived severity of COVID-19 and avoiding public transportation).

**Table 5.** Association of perceived COVID-19 susceptibility and severity with five behavioral outcomes (*N* = 450)

Perceived susceptibility and severity	Avoiding public transport (*N* = 334)		Avoiding public places (*N* = 334)		Wearing a mask (*N* = 317)		Washing hands (*N* = 64)	
	OR (95% CI)	AOR (95% CI)	OR (95% CI)	AOR (95% CI)	OR (95% CI)	AOR (95% CI)	OR (95% CI)	AOR (95% CI)
High likelihood of getting infected^a^	NR	NR	0.48 (0.27-0.87)*	0.53 (0.29-0.97)*	NR	NR	NR	NR
High likelihood of family getting infected^b^	NR	NR	NR	NR	NR	NR	3.63 (1.89-6.98)***	3.60 (1.86-6.99)***
High perceived severity of COVID-19^c^	2.00 (1.02-3.90)*	1.81 (0.91-3.59)	NR	NR	NR	NR	NR	NR
Low likelihood of survival if infected^d^	NR	NR	NR	NR	1.89 (1.04-3.40)*	1.87 (1.02-3.41)*	2.01 (1.09-3.69)*	2.01 (1.08-3.73)*

Abbreviations: AOR, adjusted odds ratios adjusted a priori for age, sex, and educational level; CI, confidence interval; NR, not reported as only statistically significant ORs are reported; OR, unadjusted odds ratios.

a A total of 56 responders self-identified as very likely or likely to get infected with SARS-CoV-2.

b A total of 52 responders identified their family as very likely or likely to get infected with SARS-CoV-2.

c A total of 412 responders perceived COVID-19 to have very high or high severity.

d A total of 81 responders self-identified as having very low or low chances of surviving COVID-19.

* *P* < 0.05.

*** *P* < 0.001.

## Discussion

The present study provides insight on perceived threats of COVID-19 and preventive measures adopted by people living in Afghanistan. While the majority of people thought that COVID-19 was a severe disease, most of them perceived themselves as less susceptible to disease. Yet, despite the lower degree of perceived susceptibility to the virus, the practice of preventive behaviors was found in most responders. Participants who believed they had a low probability of survival if infected with COVID-19 were more likely to wear a mask and practice hand washing.

Our assessment scores revealed that perceived susceptibility toward COVID-19 was relatively low while perceived severity was very high among the studied population. Given that a predominant proportion of the participants were aged under 30 years old, this finding was surprising, because previous evidence suggests relatively high transmissibility of SARS-CoV-2 and relatively low mortality in this age range.^14–16^ In the study by Atchison et al., the level of worry about the COVID-19 outbreak in the United Kingdom was 77.4%, perceived susceptibility was 47.5%, and perceived severity was 56.9% (moderate).^17^ Furthermore, perceived susceptibility and severity of SARS-CoV-2 infection were higher compared with studies regarding different pathogens such as H7N9 in urban China and SARS in Hong Kong.^18,19^ A previous study has highlighted that socio-cultural context could influence people’s perceptions toward diseases.^20^ The socioeconomic context of the mentioned countries varies greatly, which might have resulted in the difference in study findings. Therefore, this should be taken into consideration while interpreting the present findings.

Despite having low perceived susceptibility toward the virus, the majority of the population in our study followed preventive behaviors, such as avoiding places where the transmission of the virus frequently takes place, wearing a mask, and practicing hand hygiene. It could be possible that the confidence of the participants in their preventive measures might have lowered their perceived risk of infection. Moreover, the government had implemented strict containment measures, such as universities and school suspension, Nowruz restriction (Persian new year that is originally celebrated in Balkh), travel bans, the shutdown of restaurants and all entertainment/recreational facilities, and active surveillance (self-isolation and quarantine, strict confirmed case monitoring, case isolation).^12^ These containment measures might have provided them with a sense of security. More importantly, the majority of the participants in our study had comparatively higher schooling levels than the general population in Afghanistan. Educated people are more likely to follow precautionary measures and stay up to date with the epidemiological situation.

In our study, we did not find an association between perceived COVID-19 susceptibility and severity and some preventive behaviors like avoiding public transportation and restaurants, etc. These behaviors might have been influenced by the government containment measures implemented in the country. Although the Afghan government implemented diverse containment measures and active surveillance for COVID-19, this study did not specifically consider those measures as a probable variable affecting the outcome. Notably, measures taken by the government forced people to follow the rules in many countries. For example, in China, the use of face masks was widely practiced because of strict government rules.^21^ However, in the United Kingdom, despite the government’s recommendation to implement social distancing, people were reluctant to comply due to low perceived disease severity and susceptibility.^17^ Therefore, efforts should be made by individuals, as well as by the government, to mitigate the effect of a virus.

The Internet was the most commonly used source of information in our study. This finding is similar to the one from the study conducted by Cinelli et al. where social media, such as Twitter, Instagram, YouTube, Reddit, and Gab, were used mostly by participants to receive information about COVID-19.^22^ Considering their wide use, the government of Afghanistan could use these platforms to educate people about the risk of the disease and use them for critical planning of COVID-19 control measures. However, while promoting the use of the Internet, major attention should be given to the credibility of the information sources and people must be informed as to how to use those media wisely.^23^ News circulated about rural community people who tried using antibiotics, while urban people denied having symptoms due to the fear and stigma surrounding the disease. Therefore, proper education should be provided to the local community people.^24,25^

Furthermore, the spread of COVID-19 in Afghanistan is complicated by a diverse set of problems, including the influx of Afghani refugees who continue to return from Iran due to Iran’s deadly coronavirus outbreak and subsequent economic stagnation.^12^ Another concern is the low public awareness of COVID-19 and low health literacy. Indeed, cultural norms like hugging, community gatherings in religious places are still allowed. Aiming for containment may not be possible for low-income countries like Afghanistan, which cannot provide incentives for the people. The diagnostic kits shortage, lack of local laboratories, and lack of health-care workers in rural areas are other concerns. Only 9.4 skilled health-care workers and 1.9 physicians are available per 10,000 population. Due to the scarcity of doctors at the national level, health-care workers are disproportionately distributed across the country with 7.2 physicians per 10, 000 in urban areas and 0.6 in rural areas, causing considerable delays in delivering essential health interventions.^12^ While there are numerous problems (associated with health-care delivery, political instability, potential disaster, and economic hardship),^26^ multiple international organizations have been providing support to mitigate the problems associated with COVID-19.^27^ Policy-makers should work efficiently to deal with the crisis by training health workers, mobilizing them effectively, establishing laboratories, and providing essential equipment/supplies to expand testing across the country. Most importantly, the strategic vision for risk communication and community engagement (RCCE)^24^ that has been developed should be properly implemented to improve the health-care delivery system of the country.

## Limitations and Future Perspectives

The study has several limitations that should be considered. First, our study may not be generalizable to the entire population of Afghanistan. We used convenience sampling, where we received responses from a relatively younger population that only represents around 65% of the total demographics of Afghanistan.^28,29^ Therefore, responses related to perceived health status where 95.6% of participants mentioned having “good health status” should be interpreted cautiously. Moreover, we used an online survey, which might have limited the participation from older and disadvantaged groups of the population. However, although Internet users were estimated to be comparatively few, use of the Internet is an increasing trend.^30^ Second, there might have been overreporting of preventive measures, as we did not use any scale to measure the authenticity of their response. However, confidentiality and anonymity were explained in detail before data collection. Future studies could use telephone or face-to-face in-depth interviews to gain detailed insights and investigate specific aspects of the interventions promoted by health authorities.

## Conclusions

While the perceived susceptibility to SARS-CoV-2 infection was very low, the perceived severity was very high among the study population. Overall, the practice of nonpharmaceutical interventions was satisfactory, suggesting that the COVID-19 pandemic produced specific community behavioral responses in Afghanistan. The findings of this study should contribute to timely behavioral assessment of the community and are useful to design preventative interventions and risk communication strategies during the pandemic.

## Data Availability

All study instruments used on this analysis are available to researchers upon reasonable request.
